# Morphological correlation between caloric tests and vestibular hydrops in Ménière's disease using intravenous Gd enhanced inner ear MRI

**DOI:** 10.1371/journal.pone.0188301

**Published:** 2017-11-30

**Authors:** Ji Eun Choi, Yi-Kyung Kim, Young Sang Cho, Kieun Lee, Hyun Woo Park, Sung Hoon Yoon, Hyung-Jin Kim, Won-Ho Chung

**Affiliations:** 1 Department of Otorhinolaryngology—Head and Neck Surgery, Dankook University Hospital, Cheonan, Republic of Korea; 2 Department of Radiology, Samsung Medical Center, Sungkyunkwan University School of Medicine, Seoul, Republic of Korea; 3 Department of Otorhinolaryngology—Head and Neck Surgery, Samsung Medical Center, Sungkyunkwan University School of Medicine, Seoul, Republic of Korea; 4 Department of Otorhinolaryngology—Head and Neck Surgery, Gyeongsang National University Hospital, Jinju, Republic of Korea; 5 Hearing Research Laboratory, Samsung Medical Center, Seoul, Republic of Korea; Tokai University, JAPAN

## Abstract

The purpose of this study was to prove the hypothesis that caloric response in Ménière's disease (MD) is reduced by hydropic expansion of the vestibular labyrinth, not by vestibular hypofunction, by evaluating the correlation morphologically using an intravenous Gadolinium (IV-Gd) inner ear MRI. In study I, the prevalence of abnormal video Head Impulse Test (vHIT) results among the patients with definite unilateral MD (n = 24) and vestibular neuritis (VN) (n = 22) were investigated. All patients showed abnormal canal paresis (CP) (> 26%) on caloric tests. The prevalence of abnormal vHIT in patients with abnormal CP was significantly lower in MD patients (12.5%) than that in VN patients (81.8%) (*p* < 0.001). In study II, morphological correlation between caloric tests and vestibular hydrops level was evaluated in unilateral MD patients (n = 16) who had normal vHIT results. Eleven patients (61%) had abnormal CP. After taking the images of IV-Gd inner ear MRI, the vestibular hydrops ratio (endolymph volume/total lymph volume = %VH) was measured. In addition, the relative vestibular hydrops ratio (%RVH = (%VHaffected ear—%VHunaffected ear) / (%VHaffected ear + %VHunaffected ear)) was calculated. Each ratio (%VH and %RVH) was compared with average peak slow phase velocity (PSPV) and CP, respectively. In the MD patients, %VH of the affected ear correlated significantly with mean PSPV on the same side (rs = -0.569, *p* = 0.024), while %RVH correlated significantly with CP (rs = 0.602, *p* = 0.014). In most MD patients (87.5%) compared to VN patients, vHIT results were normal even though the caloric function was reduced. In addition, the reduced caloric function with normal vHIT was related to the severity of the vestibular hydrops measured by the IV-Gd inner ear MRI. These findings concluded that the abnormal caloric tests with normal vHIT in MD indicated severe endolymphatic hydrops rather than vestibular hypofunction.

## Introduction

Ménière's disease (MD) is an idiopathic inner ear disorder with symptom complex of recurrent vertigo, hearing loss, tinnitus, and ear fullness. Although the natural course of MD varies highly, most patients suffer repeated vertigo attacks and gradually lose their hearing and vestibular function [1]. The endolymphatic hydrops is currently known as a histologic hallmark of MD, but the causes of MD are unknown [2].

Caloric test has been used to assess the vestibular function in MD. Bithermal water irrigation into the external ear canal causes the convective current in the endolymph of the horizontal semicircular canal (HSCC) to induce the cupular deflection [3]. Resulting peak slow phase eye velocity (PSPV) was measured and canal paresis (CP) was calculated by comparing PSPV from both sides which indicates the function of vestibule-ocular reflex (VOR) at low frequency (0.002–0.004 Hz). In about half the cases of unilateral MD, canal paresis (CP) on the affected side was reported [4,5].

Recently, video head impulse test (vHIT) has been introduced to evaluate VOR at high frequency (4–7 Hz) [6]. In contrast to the caloric tests, the vHIT is a more physiologic test of the HSSC for assessing the high-frequency angular VOR [6]. Interestingly, in several studies, the prevalence of abnormal vHIT results in MD patients was relatively small compared to the reduced caloric response [7,8]. To explain such dissociation between the caloric test and vHIT in MD, two hypotheses have been proposed. One was the selective damage of low frequency specific vestibular end organs. The other was that the caloric response was reduced by mechanical reason, rather than true vestibular hypofunction.

The first hypothesis that hair cells were selectively damaged in frequency-specific way was supported by several studies [5,9]. Two types of hair cells (type 1 and 2) in the vestibular end organs exists and physiologically, type 1 hair cells are to be synapsed with irregular afferents associated with high acceleration stimuli, while type 2 hair cells are synapsed with regular afferents associated with low acceleration stimuli [10,11]. Based on the post mortem histological studies of the human temporal bone, it is assumed that type 2 hair cells are more selectively damaged than type I hair cells during the progress of MD [9]. Since the caloric test uses a non-physiologic and low frequency stimulation compared to vHIT, loss of type II hair cells would reduce caloric response, while the intact type I hair cells were more likely to preserve VOR responses during vHIT [10–12]. However, in a recent study, similar degrees of degeneration in both type I and type II hair cells have been found using fresh tissue from inner ears of living human [13]. Furthermore, both type I and type II hair cells can provide signals to both regular and irregular afferent neurons, which means there is no simple correlation between hair cell types and afferent fiber types [14].

The second hypothesis that the caloric response was not reduced by the true vestibular hypofunction was suggested by several reports for the dissociation of caloric and vHIT test [12,15]. The dissociation of prevalence of vHIT and caloric tests in MD states that the endolymphatic hydrops in the HSCC is responsible for the reduced caloric response without losing physiologic VOR response. Hydropic ears in the HSCC can permit convective recirculation of fluid within the duct and allow dissipation of hydrostatic force induced by the caloric test, which is not the case in normal HSCC. However, this theory has not been confirmed yet morphologically.

Recently, MR imaging has been used to visualize endolymphatic hydrops in living MD patients by either intratympanic or intravenous gadolinium (IV-Gd) injection [16–19]. There were several reports investigating the relationship between hydrops level in MR imaging and CP on caloric tests. Gurkov et al. found a correlation between hydropic expansion into the HSCC and CP on caloric tests [20]. However, the other studies reported that there was no correlation between CP and the vestibular hydrops level [21,22].

The prospective study is designed to prove this second hypothesis that caloric response was reduced by physical enlargement of membranous duct in the hydropic labyrinth in MD, not by vestibular hypofunction. The first part was to demonstrate the dissociation of CP and vHIT in MD patients who had acute vertigo attacks. The second part was to evaluate the relationship between caloric response and vestibular hydrops level in unilateral MD patients using MR imaging after an IV-Gd administration.

## Methods

### Ethics statement

Study I was performed retrospectively and Study II was performed prospectively. All participants in Study II provided written informed consent before completing the study. Approval for this research studies including retrospective and prospective study were obtained from the Institutional Review Board of Samsung Medical Center (IRB No. 2014-08-101-007).

### Subjects

#### Study I

Medical records of all patients who visited the dizziness clinic between May 2015 and June 2016 were reviewed retrospectively. Inclusion criteria were as follows: (1) Patients who had history of vertigo within the last 3 months; (2) Patients who were diagnosed as definite unilateral MD according to the American Academy of Otolaryngology-Head and Neck Surgery (AAO-HNS) criteria (1995) [23] or unilateral VN according to the Cooper’s criteria (1993) [24]; (3) Patients who were tested for both bithermal caloric test and vHIT on the same day; (4) Patients who showed unilateral vestibular weakness (CP >26%) based on the bithermal caloric test.

#### Study II

Patients with definite MD, according to the AAO-HNS criteria (1995) [23], were prospectively recruited for MR imaging between December 2015 and June 2016 to visualize the endolymphatic hydrops. Inclusion criteria were as follows: (1) Patients who had two or more spontaneous episodes of vertigo, each lasting more than 20 minutes during the last 3 months; (2) Patients who had unilateral ear symptom of tinnitus, hearing loss, or ear fullness. All patients underwent MR scanning 4 hours after intravenous administration of Gadobutrol (gadolinium-DO3A-butriol, Gadovist 1.0; Schering, Berlin, Germany) to evaluate the degree of endolymphatic hydrops. Caloric test and vHIT were also performed on the same day.

We excluded patients who were treated with intratympanic gentamicin injection or endolymphatic sac decompression. We also excluded patients who showed abnormal vHIT results (low gain with saccade) for the horizontal canal, in case these patients had vestibular dysfunction with VOR loss.

### Vestibular function tests

Caloric test was performed using an infrared video-oculographic system (Micromedical Technologies, Chatham, Illinois, USA) and a Brookler–Grams closed-loop irrigation unit. Each ear was irrigated alternately with constant flow of water at temperatures of 30 and 44°C for 40 seconds. Peak slow phase velocity (PSPV) of nystagmus was measured following each irrigation. Jongkees’ formula (1962) [25] was used to determine canal paresis (CP). If CP was >26%, the result was considered indicative of abnormal CP.

The vHIT was performed using the ICS impulse (GN Otometrics, Taastrup, Denmark). Subjects were seated wearing goggles facing the wall 1 m away from a small LED target placed on the wall. Subjects were instructed to stare at the fixation dot while the examiner standing behind the subject rotated the participant’s head in a brief, abrupt, and unpredictable manner. To test each semicircular canal, about 20 head impulses in each direction were manually delivered at a small amplitude (5~15 degrees) with high peak velocity (150~250 degrees per second). To test the horizontal semicircular canal, the head was rotated to the right and left in the horizontal plane. To test the anterior and posterior semicircular canals, head rotations were delivered in vertical, left anterior-right posterior (LARP), and right anterior-left posterior (RALP) planes. Participants’ heads were rotated up or down in the sagittal plane while their heads were tilted about 45° to the left to test the RALP plane or 45° to the right to test the LARP plane. Catch up saccades were defined according to their appearance as covert (during the head impulse) and overt (once the head impulse ended). They were taken into consideration if the velocity of the saccade was above 50°/s. Abnormal result was defined when there was low gain (< 0.8 for the horizontal canal and < 0.7 for the vertical canals) with saccades according to a previous study using normal subjects [26,27]

### MR imaging

MR imaging was performed on a 3.0-tesla unit (MAGNETOM Skyra; Siemens Medical Solutions, Erlangen, Germany) using a 32-channel array head coil. All patients underwent heavily T2-weighted MR cisternography (MRC) for anatomical reference of the total lymph fluid, hT2W-3D-FLAIR with inversion time of 2250 ms (positive perilymph images; PPI), and 2050 ms (positive endolymph images; PEI) to generate HYbriD of Reversal image Of Positive endolymph signal nature image of positive perilymph signal (HYDROPS) images. Detailed parameters for MRC, PPI, and PEI have been described accordingly [19,22]. HYDROPS images were generated on the scanner console by subtracting PEI from PPI. Generated HYDROPS images were saved and transferred to a PACS server as a separate series. Negative signal values in subtraction results were allowed. To increase the contrast-to-noise ratio (CNR) of the HYDROPS images, HYDROPS-Mi2 images were generated on a DICOM viewer (OsiriX MD image software, ver. 7.5.1 64 bit; Pixmeo Sarl, Bernex, Switzerland) as described previously [28]. HYDROPS and MRC images were transferred from a CD-ROM to a Mac-Book personal computer (Apple Computer, Inc., Cupertino, CA, USA) with OsiriX MD software which allowed easy pixel-by-pixel multiplication of HYDROPS and MRC images within a few seconds.

### Imaging analysis

Two observers (with 7 years’ experience in neuroradiology and 5 years’ experience in otology, respectively) independently evaluated images as described previously [29]. Each observer manually contoured the vestibule separately to set up a region of interest (ROI) on MRC based on the following instructions: (1) set the image window level and width to 400/1000 before starting the contouring of the vestibule on MRC; (2) select the lowest slice where the lateral semicircular canal ring is visualized more than 240°; (3) exclude semicircular canal and ampulla when drawing the ROI for the vestibule on MRC. These ROIs drawn on MRC were copied and pasted onto HYDROPS-Mi2 images. Both observers then used the histogram function of the OsiriX MD software to measure the numbers of all the pixels in the ROI and the numbers of pixels with negative signal intensity values (i.e., endolymph) in the ROI.

The vestibular hydrops ratio (%VH) was defined as the ratio of the area (%) of the endolymphatic space in the entire lymphatic space in the vestibule. The vestibular hydrops ratio was calculated with the following equation: %VH = (number of negative pixels for the endolymph in the ROI / total number of pixels in the ROI) x 100. The vestibular hydrops ratio was measured for both ears separately by the two observers. To correlate with CP, relative vestibular hydrops ratio (%RVH) was calculated similar to Jongkees’ formula [25]: %RVH = (%VHaffected—%VHunaffected) / (%VHaffected + %VHunaffected).

### Statistical analysis

All data were analyzed using SPSS 18.0 (SPSS Inc., Chicago, IL, USA). Mean CP values were compared using the unpaired t-test for study I. To compare the prevalence of abnormal response of vHIT between patients with MD and VN for study I, the Chi-square test was used.

Inter-observer reliability was calculated using the Kendall’s tau b correlation coefficient for study II. To compare the vestibular hydrops ratios between affected and unaffected ears for study II, paired-t test was used. Morphologic correlation between caloric results and vestibular hydrops was evaluated in two ways using the spearman correlation analysis. First, caloric response was defined as average PSPV of nystagmus following warm and cold irrigation on the same side. The association between caloric response and the vestibular hydrops ratio (%VH) was analyzed. Second, we assigned positive and negative CP values to differentiate between affected ear and unaffected ear. Positive results of CP meant paresis on the affected ear while negative results of CP meant paresis on the unaffected ear. The association between CP values and relative vestibular hydrops ratio (%RVH) was analyzed.

## Results

### Study I: Dissociation of video head impulse test (vHIT) and caloric test

A total of 46 patients were enrolled, 24 patients with MD and 22 patients with VN. Demographic characteristics are shown in S1 and S2 Tables. There was no significant difference of demographic characteristics such as sex, age, affected side, CP on caloric testing between two groups. The mean value of CP was 55% ± 19.8% in MD patients and 65.5% ± 21.1% in VN patients. One patient with MD showed unilateral weakness on the opposite side rather than the affected side. Regardless of the side of the weakness, the CP values of MD patients were not significantly different from those of VN patients (*t* = 1.745, *df* = 44, *p* = 0.088, unpaired t-test). However, the prevalence of abnormal vHIT results was significantly lower in patients with MD (3/24 = 12.5%) compared to that in patients with VN (18/22 = 81.8%) (*p* < 0.001, Chi-square analysis).

### STUDY II: Morphological correlation between canal paresis (CP) and vestibular hydrops in MD using inner ear MR imaging

A total of 16 patients with definite unilateral MD with normal vHIT test underwent MR scanning. Demographic characteristics such as sex, age, affected side, pure tone thresholds, results of the caloric tests, and vestibular hydrops ratios are shown in Table 1. Of the 16 patients, 11 (61%) showed unilateral vestibular weakness (CP >26%) based on caloric testing. Among these patients, one patient (S11) showed abnormal CP on the opposite side (Table 1).

**Table 1:** Demographic characteristics of patients with definite unilateral Ménière's disease in Study II.

Subject	Sex	Age	MD Side	PTA* (dB)	PSPV^¶^		Caloric test		Vestibular hydrops (%)		HSCC involvement	
					affected side	unaffected side	weaker side	CP (%)	affected side	unaffected side	affected side	unaffected side
S1	F	31	R	51.3	0	18	R	42	79%	15%	(-)	(-)
S2	M	63	R	66.3	12.5	20.5	R	24	83%	17%	(-)	(-)
S3	M	61	L	68.8	14.5	28.5	L	33	75%	13%	(-)	(+)
S4	M	21	R	22.5	34.5	26	L	14	29%	19%	(-)	(-)
S5	M	62	R	58.8	18.5	42.5	R	39	57%	3%	(-)	(-)
S6	M	53	R	72.5	2	7.5	R	58	100%	6%	(+)	(+)
S7	M	53	L	78.8	4.5	12	L	45	92%	9%	(+)	(+)
S8	F	46	R	50.0	8	16	R	33	93%	23%	(+)	(-)
S9	M	46	L	30.0	7.8	10.1	L	13	52%	21%	(-)	(-)
S10	F	34	L	35.0	2	7	L	50	84%	39%	(+)	(-)
S11	M	27	R	41.3	15.5	4.5	L	55	91%	83%	(+)	(+)
S12	M	65	R	55.0	19	19	R	0	71%	6%	(-)	(-)
S13	M	52	L	72.5	4.5	15.5	L	55	83%	1%	(+)	(-)
S14	M	56	R	71.3	3.5	17	R	66	86%	3%	(+)	(-)
S15	M	54	R	57.5	17	26	R	21	26%	10%	(-)	(+)
S16	F	43	L	10.0	9	19.5	L	24	60%	18%	(+)	(+)

R: right; L: left; CP: canal paresis; HSCC: horizontal semicircular canal; PTA*: average pure tone thresholds for 4 frequencies (0.5, 1k, 2k, and 4k Hz); PSPV^**¶**^: average of the peak slow phase velocity of the warm and cold irrigation

**Fig 1:**
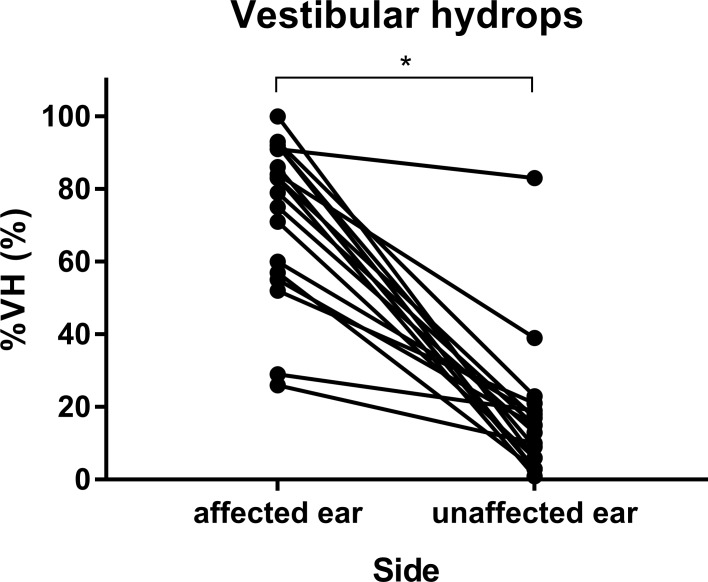
Vestibular hydrops ratio in affected and unaffected ears. Vestibular hydrops ratios (%VH) were measured to be 72.5% ± 21.8% in affected ears and 16.9% ± 19% in unaffected ears. An asterisk (*) indicates significant difference.

Inter-observer reliability of vestibular hydrops ratio resulted in Kendall’s tau coefficient of 0.767 for affected ears and 0.946 for unaffected ears, indicating high inter-observer reliability. The vestibular hydrops ratios for affected and unaffected ears of 16 MD patients are shown in Fig 1. The mean vestibular hydrops ratios of affected and unaffected ears were 72.5 ± 21.8% and 16.9 ± 19%, respectively. Vestibular hydrops was more severe in the affected ear than the unaffected ear (*t* = 8.771, *df* = 17, *p* < 0.001, Fig 1).

$$\%VH=\frac{number\;of\;negative\;pixels\;for\;the\;endolymph\;in\;the\;ROI\;}{total\;number\;of\;pixels\;in\;the\;ROI}\;\times 100$$

**Fig 2:**
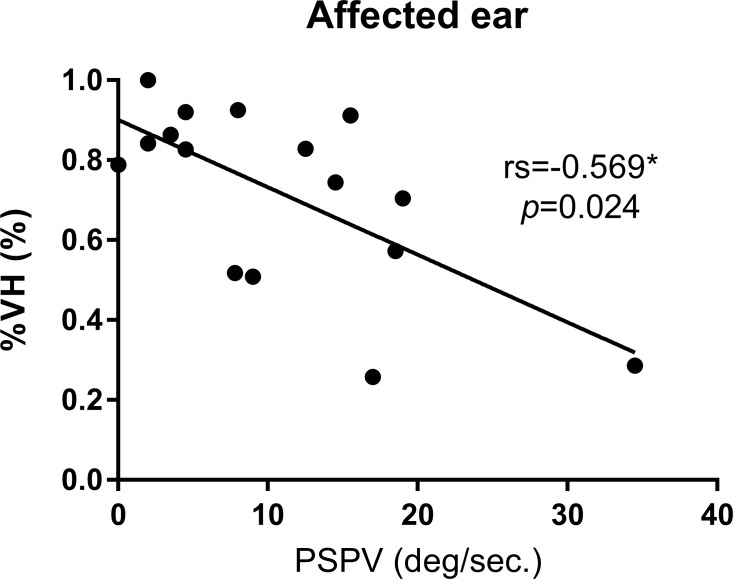
Scatter plots of the vestibular hydrops ratio and caloric response. X-axis represents caloric response which was defined as the average of peak slow phase velocity (PSPV) of nystagmus following warm and cold irrigation in each ear, while Y-axis represents the vestibular hydrops ratio. Results of scatter plots are shown in circle (●) for affected ears. rs, Spearman correlation coefficient; p, significance. An asterisk (*) indicates significant relationship.

To examine our hypothesis, as to which degree caloric loss correlates with the severity of vestibular hydrops rather than the degree of vestibular dysfunction, the morphological correlation between caloric response and the severity of vestibular hydrops was evaluated by excluding the patients who showed abnormal VOR based on vHIT. The relationship between vestibular hydrops ratio (%VH) and caloric response for the affected ear is shown in Fig 2. The vestibular hydrops ratio of the affected ear correlated significantly with the caloric response (average PSPV) (rs = -0.569, p-value = 0.024).

**Fig 3:**
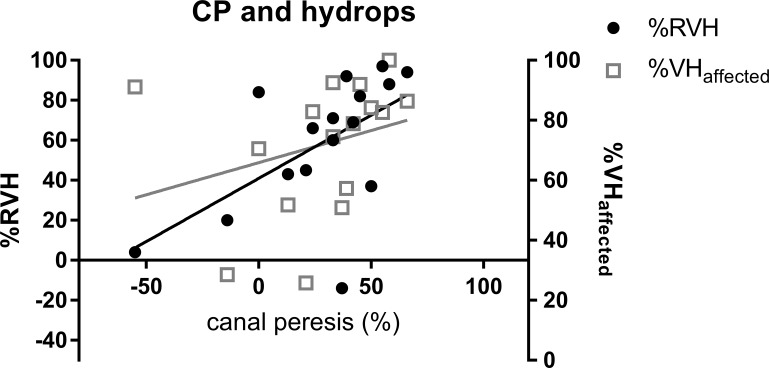
Scatter plots of vestibular hydrops ratio (%VH) and relative vestibular hydrops ratio. X-axis represents canal paresis (%) while Y-axis represents the vestibular hydrops ratio and relative vestibular hydrops ratio. Results of scatter plots are shown in square (□) for vestibular hydrops ratio (%VHaffected) and in circle (●) for relative vestibular hydrops ratio (%RVH). The value of CP correlated significantly with relative vestibular hydrops ratio (*rs* = 0.602, *p* = 0.014), but not with the vestibular hydrops ratio (*rs* = 0.468, *p* = 0.068).

Correlation between the CP value and the vestibular hydrops ratio (%VH) of the affected ear and relative vestibular hydrops ratio (%RVH) are shown in Fig 3. CP value correlated significantly with %RVH (*rs* = 0.602, *p* = 0.014), but not with %VH (*rs* = 0.468, *p* = 0.068).

$$\%VH=\frac{number\;of\;negative\;pixels\;for\;the\;endolymph\;in\;the\;ROI\;}{total\;number\;of\;pixels\;in\;the\;ROI}\;\times 100$$

$$\%RVH=\frac{\%VH_{affecte}\;-\;\%VH_{unaffected}}{\%VH_{affected}+\;\%VH_{unaffected}}$$

## Discussion

In Study I, we found that even though the caloric function was reduced, the high frequency VOR function revealed by vHIT was preserved in most patients with MD (87.5%). In addition, caloric results correlated with vestibular hydrops level measured by IV-Gd inner ear MRI in Study II. These findings support the hypothesis that the caloric response in MD patients were affected by the physical enlargement of the membranous labyrinth by the hydropic change rather than true vestibular dysfunction [30].

Assessments of vestibular function in MD patients were important for several reasons. It not only documents the amount of vestibular damage accumulated during the natural course of MD, it can also be used for differential diagnosis of other disease such as VN, labyrinthitis and superior canal dehiscence syndrome. In addition, monitoring the functional vestibular status is important when deciding on destructive treatments such as labyrinthectomy, vestibular neurectomy and intratympanic gentamicin treatment.

To assess the vestibular functions in MD patients, the caloric test has been used for a long time. Throughout the natural course of Meniere’s disease (MD), repeated vertigo attacks with fluctuating hearing loss, and during the final stage of MD, hearing threshold about 40–50 dB due to sensorineural hearing loss and 50% caloric loss is reported [1]. In other words, patients progressively lose their hearing and vestibular function by repeated attacks, and resulted in persistent non-whirling type of dizziness with fixed sensorineural hearing loss. However, in a clinical situation, even if the caloric function was reduced, the patients still suffered from recurrent whirling type vertigo attacks with the same initial episodes, which indicates the vestibular function was still preserved.

Furthermore, there were many studies reporting dissociation of test results between caloric test and vHIT in MD [5,7,12,30]. Halmagyi et al. [12] have proposed a new mechanism for explaining the dissociation between the caloric response and vHIT result (the so called ‘hydrostatic temperature dissipation’). In healthy ears, the thermal stimulus in the caloric test causes the hydrostatic pressure to induce a cupular deflection. However, like MD patients, if the hydropic expansion of the duct allows local flow within the duct, it will dissipate the hydrostatic pressure caused by the thermally-induced density difference and diminish or eliminate the deflection of the cupula. Thus, the reduced caloric response in MD was due to the hydropic expansion of endolymphatic duct without compromising the VOR responses in vHIT.

To prove this hypothesis morphologically, study II was designed to evaluate the morphological correlation between the caloric response and the degree of vestibular hydrops in unilateral MD patients using an inner ear MRI. Previously, a few have studied the relationship between the caloric response and the hydrops in MD using inner ear MRI but didn’t show any significant relationship between the caloric response and the degree of endolymphatic hydrops [21,22]. However, Gurkov et al. demonstrated that the endolymphatic hydrops in the semicircular canal correlated with the reduced caloric function [20].

**Fig 4:**
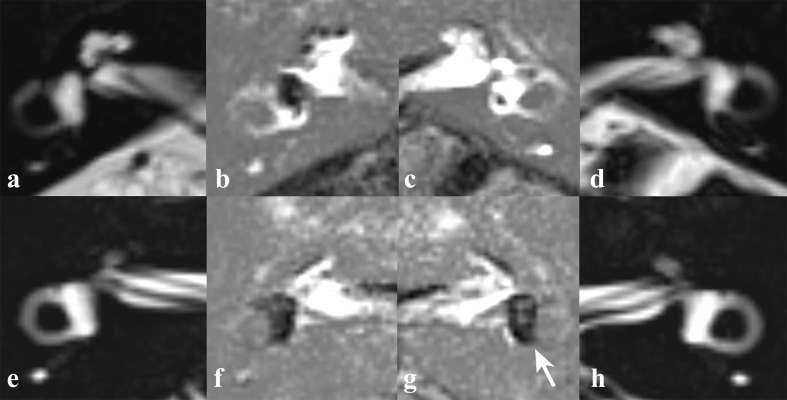
MR cisternography (MRC) and HYDROPS2 imaging of two unilateral MD patients. MRC demonstrates regular anatomy of fluid filled spaces of the inner ears of subject 5 (a, d) and subject 11 (e, h). Both subject 5 (a-d) and 11 (e-h) have unilateral ear symptom on the right side and vestibular hydrops are observed in the affected ears (b, f). Additionally, asymptomatic vestibular hydrops in the affected side is observed in subject 11 (g, arrow).

In our study, we clearly demonstrated the correlation between the caloric response and the vestibular hydrops level. The main difference from previous studies was the inner ear MRI technique and the patients group. Previous studies used inner ear MRI using intratympanic Gd injection. Therefore, the hydrops level was retrieved from the affected ear only, which is a problem since CP on the caloric test is a comparative ratio between both ears. In addition, MD has a chance of bilaterality up to 75% [1,31]. Even though there were no symptoms, there might have been endolymphatic hydrops in the unaffected ear (Fig 4). Therefore, it seems inappropriate to compare CP with the hydrops level in the affected side only. We used IV-Gd inner ear MRI which can visualize both inner ears simultaneously. The vestibular hydrops level was measured from both ears. CP was compared to both vestibular hydrops levels (%VH) from the affected side and the relative vestibular hydrops level ratio (%RVH) to reflect the both sides. As a result, the correlation between CP and the relative vestibular hydrops level was statistically significant (rs = 0.602, p-value = 0.014). But the correlation between CP and the vestibular hydrops level in the affected side was not statistically significant similar to other reports (rs = 0.468, p-value = 0.068) (Fig 3). In contrast, the caloric response expressed by average PSPV on the affected side correlated significantly with the vestibular hydrops ratio on the same side (rs = -0.569, p-value = 0.024, Fig 2). These findings demonstrated that the severity of vestibular hydrops could affect the caloric response by reducing PSPV (i.e. reducing the cupular deflection by the dissipation of hydrostatic drive in the hypothesis).

In addition, unlike previous studies, we excluded the patients with abnormal vHIT, history of previous intratympanic gentamicin injection and surgical treatment. Because these patients might actually have vestibular hypofunction. These patients were excluded to assess the correlation between the caloric response and the hydropic level of labyrinthine duct in the patients with normal VOR function.

**Fig 5:**
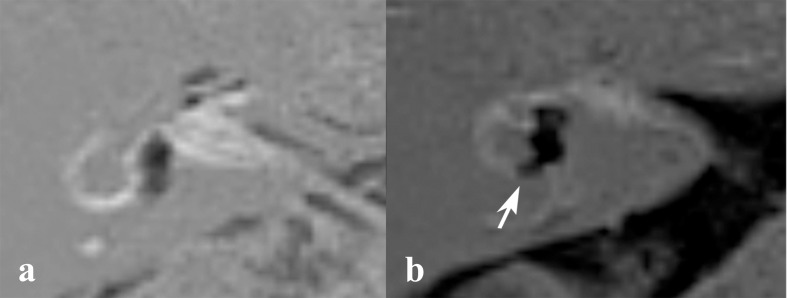
HSCC involvement on HYDROPS2 imaging. Both subject 2 (a) and 8 (b) have unilateral ear symptom on the right side. Subject 8 has a hydropic expansion of the horizontal canal (b, arrow) while subject 2 dose not (a).

This study had shortcomings. The hydrops ratio was measured in the vestibule rather than the HSCC, which was attributed to technical issues. The vestibule is the largest among the cochlea, vestibule, semicircular canal in the inner ear, and the most reliable area to check the hydrops level by inner ear MRI with our method. In addition, the pathogenesis in MD began from saccule in the vestibule, and extended to the semicircular canal. Hydropic involvement of semicircular canals might be related to disease progression and the severity of vestibular hydrops [20,32]. In our study, the hydropic expansions of horizontal canal were observed in 8 cases for affected ears out of 16 MD patients (Table 1 and Fig 5). In our data, cases which showed hydropic involvement of horizontal canal had significantly higher vestibular hydrops ratio (%VH) compared to cases without such morphologic feature (%VH for ears involved horizontal canal: 85.0 ± 14.8%; %VH for ears without involvement of horizontal canal: 58.8 ± 22.1%, P = 0.007 in Mann-Whitney analysis) (Table 1). In addition, the caloric response in cases with hydropic involvement of horizontal canal was significantly lower than cases without such morphologic feature among MD patients who showed normal VOR response in vHIT (average PSPV for cases involved horizontal canal: 6.1 ± 4.6°/sec; average PSPV for cases without involvement of horizontal canal: 15.5 ± 9.9°/sec, P = 0.046 in Mann-Whitney analysis) (Table 1). With these data, even though we didn’t find direct correlation between the caloric response and hydrops level in the HSCC, the vestibular hydrops ratio would represent the severity of hydrops in the vestibule as well as the semicircular canal.

In conclusion, most patients with unilateral MD tended to have normal VOR in vHIT even though they had abnormal CP in caloric tests. To explain this dissociation, IV-Gd-inner ear MR imaging revealed that caloric test was associated with the severity of hydrops in the vestibule. Therefore, abnormal CP in MD in normal vHIT is more likely to indicate severe hydrops rather than loss of vestibular functions.

## Supporting information

S1 Table Demographic characteristics of patients with definite unilateral Ménière's disease in Study I. (DOCX)

S2 Table Demographic characteristics of patients with unilateral vestibular neuritis in Study I. (DOCX)
