# Supplementary material for: Morphological correlation between caloric tests and vestibular hydrops in Ménière's disease using intravenous Gd enhanced inner ear MRI
Source: PLoS One. 2017 Nov 30;12(11):e0188301. doi: 10.1371/journal.pone.0188301 (PMC5708622; doi:10.1371/journal.pone.0188301)
Supplement: S1 Table — (DOCX) [file pone.0188301.s001.docx]

**S1 Table. Demographic characteristics of Patients with Definite unilateral Ménière's disease in Study I**

| **Subject** | **Sex** | **Age** | **MD Side** | **caloric test** | | **video Head Impulse Test** | | | | | |
| --- | --- | --- | --- | --- | --- | --- | --- | --- | --- | --- | --- |
|  |  |  |  | **weaker side** | **CP (%)** | **affected side** | | | **unaffected side** | | |
|  |  |  |  |  |  | **LC** | **PC** | **AC** | **LC** | **PC** | **AC** |
| MD1 | F | 31 | R | R | 42 | NL | NL | NL | NL | NL | NL |
| MD2 | F | 59 | L | L | 58 | NL | NL | NL | NL | NL | NL |
| MD3 | M | 62 | R | R | 39 | NL | NL | NL | NL | NL | NL |
| MD4 | M | 53 | R | R | 58 | NL | NL | NL | NL | NL | NL |
| MD5 | M | 53 | L | L | 45 | NL | NL | NL | NL | NL | NL |
| MD6 | F | 75 | R | R | 100 | NL | NL | NL | NL | NL | NL |
| MD7 | F | 34 | L | L | 50 | NL | NL | NL | NL | NL | NL |
| MD8 | M | 27 | R | L | 55 | NL | NL | NL | NL | NL | NL |
| MD9 | M | 52 | L | L | 55 | NL | NL | NL | NL | NL | NL |
| MD10 | M | 56 | R | R | 66 | NL | NL | NL | NL | NL | NL |
| MD11 | F | 43 | L | L | 37 | NL | NL | NL | NL | NL | NL |
| MD12 | M | 79 | R | R | 75 | NL | NL | NL | NL | NL | NL |
| MD13 | F | 62 | L | L | 55 | NL | NL | NL | NL | NL | NL |
| MD14 | F | 59 | L | L | 42 | NL | NL | NL | NL | NL | NL |
| MD15 | M | 65 | R | R | 79 | NL | NL | NL | NL | NL | NL |
| MD16 | F | 55 | R | R | 90 | abNL | NL | NL | NL | NL | NL |
| MD17 | F | 77 | R | R | 53 | NL | NL | NL | NL | NL | NL |
| MD18 | F | 73 | R | R | 92 | NL | NL | NL | NL | NL | NL |
| MD19 | M | 55 | R | R | 32 | abNL | NL | NL | NL | NL | NL |
| MD20 | F | 56 | L | L | 45 | NL | NL | NL | NL | NL | NL |
| MD21 | F | 44 | L | L | 56 | NL | NL | NL | NL | NL | NL |
| MD22 | M | 61 | L | L | 33 | NL | NL | NL | NL | NL | NL |
| MD23 | F | 46 | R | R | 33 | NL | NL | NL | NL | NL | NL |
| MD24 | F | 44 | L | L | 29 | abNL | NL | NL | NL | NL | NL |

R: right; L: left; CP: canal paresis; LC: lateral canal; PC: posterior canal; AC: anterior canal; NL: normal; abNL: abnormal
