# Supplementary material for: Morphological correlation between caloric tests and vestibular hydrops in Ménière's disease using intravenous Gd enhanced inner ear MRI
Source: PLoS One. 2017 Nov 30;12(11):e0188301. doi: 10.1371/journal.pone.0188301 (PMC5708622; doi:10.1371/journal.pone.0188301)
Supplement: S2 Table — (DOCX) [file pone.0188301.s002.docx]

**S2 Table. Demographic characteristics of Patients with unilateral Vestibular neuritis in Study I**

| **Subject** | **Sex** | **Age** | **caloric test** | | **video Head Impulse Test** | | | | | |
| --- | --- | --- | --- | --- | --- | --- | --- | --- | --- | --- |
|  |  |  | **weaker side** | **CP (%)** | **affected side** | | | **unaffected side** | | |
|  |  |  |  |  | **LC** | **PC** | **AC** | **LC** | **PC** | **AC** |
| VN1 | M | 41 | R | 52 | NL | abNL | NL | NL | NL | NL |
| VN2 | F | 34 | L | 57 | abNL | NL | NL | NL | NL | NL |
| VN3 | F | 55 | L | 43 | NL | NL | NL | NL | NL | NL |
| VN4 | M | 55 | R | 56 | abNL | NL | NL | NL | NL | NL |
| VN5 | M | 44 | L | 100 | abNL | NL | NL | NL | NL | NL |
| VN6 | F | 36 | L | 93 | abNL | NL | NL | NL | NL | NL |
| VN7 | M | 57 | R | 100 | abNL | NL | abNL | NL | NL | NL |
| VN8 | M | 21 | R | 51 | NL | NL | NL | NL | NL | NL |
| VN9 | M | 49 | R | 77 | abNL | NL | NL | NL | NL | NL |
| VN10 | F | 57 | R | 84 | abNL | NL | NL | NL | NL | NL |
| VN11 | F | 67 | L | 65 | abNL | NL | NL | NL | NL | NL |
| VN12 | M | 46 | R | 83 | abNL | NL | abNL | NL | NL | NL |
| VN13 | M | 66 | R | 50 | abNL | NL | NL | NL | NL | NL |
| VN14 | M | 57 | L | 55 | NL | NL | NL | NL | NL | NL |
| VN15 | M | 58 | L | 56 | NL | abNL | NL | NL | NL | NL |
| VN16 | M | 75 | R | 41 | abNL | NL | NL | NL | NL | NL |
| VN17 | M | 57 | R | 64 | abNL | NL | abNL | NL | NL | NL |
| VN18 | M | 41 | L | 46 | abNL | NL | NL | NL | NL | NL |
| VN19 | F | 74 | R | 100 | abNL | abNL | abNL | NL | NL | NL |
| VN20 | F | 21 | R | 50 | abNL | NL | NL | NL | NL | NL |
| VN21 | M | 59 | L | 86 | abNL | abNL | NL | NL | NL | NL |
| VN22 | M | 33 | L | 32 | NL | NL | NL | NL | NL | NL |

R: right; L: left; CP: canal paresis; LC: lateral canal; PC: posterior canal; AC: anterior canal; NL: normal; abNL: abnormal
